# Rectangularity Is Stronger Than Symmetry in Interpreting 2D Pictures as 3D Objects

**DOI:** 10.3389/fnhum.2022.849159

**Published:** 2022-04-25

**Authors:** Kokichi Sugihara, Baingio Pinna

**Affiliations:** ^1^Meiji Institute for Advanced Study of Mathematical Sciences, Meiji University, Tokyo, Japan; ^2^Department of Biomedical Sciences, University of Sassari, Sardinia, Italy

**Keywords:** depth perception, depth illusion, shape constancy, rectangularity, symmetry, image interpretation, room-size illusion

## Abstract

It is known that the human brain has a strong preference for rectangularity in interpreting pictures as 3D shapes. Symmetry is also considered to be a factor that the human vision system places high priority on when perceiving 3D objects. Thus, a question is raised: which is more basic, the rectangularity preference or the symmetry preference? To answer this question, we carried out experiments using pictures that have at least two interpretations as 3D objects, one of which was rectangular but not symmetric, and the other of which was symmetric but not rectangular. We found that the preference for rectangularity is stronger than that for symmetry. This observation will help us to understand various 3D optical illusions, including the room-size illusion and the ambiguous object illusion.

## Introduction

Visual perception of depth is one of the fundamental functions of the human vision system because we need depth information in order to act in 3D environments, such as grasping objects and avoiding obstacles. When we see a 3D object directly, we can use two eyes and consequently the binocular stereo works to perceive the depth. When we see a projected image of a 3D object, on the other hand, perception of depth is not easy because the image is 2D, with the depth information lost. When we see an image using two eyes, the binocular stereo tells us the distance to the sheet of paper on which the image is printed, but not to the object represented in the image. Indeed, a single image does not specify the 3D shape of the object uniquely; various 3D shapes can create the same 2D projected image (Marr, 1982; Sugihara, 1986). However, we usually interpret images of simple objects such as cubes and bricks without any difficulty. This means that the human vision system does not consider all possibilities, but only a small subset of possible interpretations, determined by certain rules (Clowes, 1971; Hoffman, 1998; Hertzmann, 2020).

It has been observed that the human vision system has a strong preference for rectangular objects (Perkins, 1971, 1972, 1973). Indeed, a picture of a parallelepiped is almost always interpreted as a rectangular parallelepiped if this interpretation is mathematically possible. This property is also used effectively for machine interpretation of engineering drawings (Valley et al., 2004). Even an Ames room, which is a particular type of non-rectangular room, is perceived as rectangular from a particular viewpoint (Gregory, 1970; Ninio, 2001).

In Gestalt psychology, on the other hand, symmetry is also considered to be a factor that the human vision system places high priority on when perceiving 3D objects (Michaelsen et al., 2013; Michaelsen, 2014). Symmetry is effectively assumed in order to distinguish 3D structures from 2D pictures (Sawada et al., 2014). Indeed, a rectangular parallelepiped is highly symmetric, and such symmetric structures can explain why we perceive a cube from the Necker cube figure (Hidaka and Takahashi, 2021).

Therefore, it is not clear whether the human vision system is choosing a rectangular object or a highly symmetric object in perceiving a rectangular parallelepiped from the picture. Thus, a question is raised: which is stronger, the rectangularity preference or the symmetry preference? To answer this question is the aim of this paper. For this purpose, we generate pictures that have two interpretations, one is rectangular but not symmetric, and the other is symmetric but not rectangular. Using these pictures, we will examine which preference is stronger.

## Materials and Methods

### Pictures of Rectangular Parallelepipeds

**FIGURE 1:**
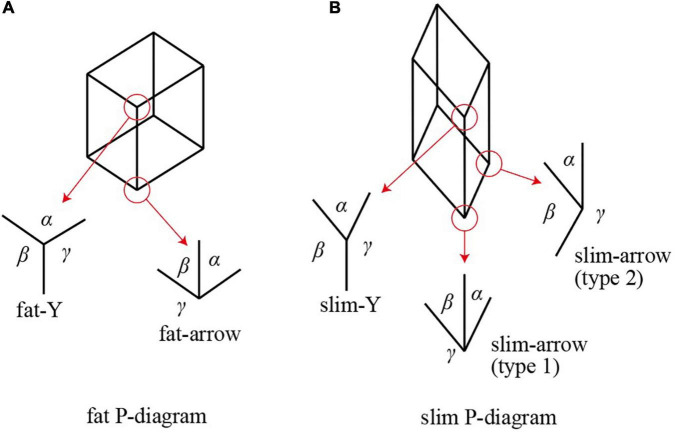
P-diagrams: **(A)** fat; **(B)** slim.

We used pictures of rectangular parallelepipeds to create our experimental materials. Suppose that a parallelepiped is projected orthogonally on the picture plane and all of the edges are drawn regardless of whether they are visible or not. We call this kind of a picture a wireframe picture. Figure 1 shows two typical examples. Each consists of eight vertices and twelve edges, connected as shown in the figure, and the edges are classified into three groups of mutually parallel lines. Let us call this kind of a diagram a *P-diagram* (an abbreviation of “parallelepiped diagram”). We assume that the projection is done from a viewpoint such that no two vertices fall on a common point and no two edges fall on a common line.

Vertices of a parallelepiped form two types of junctions in the associated P-diagram. One is a Y-shaped junction at which three edges meet and divide the surrounding space into three sectors with angles α, β, and γ all less than 180°, as show in Figure 1. The other is an arrow-shaped junction at which three edges meet and one of the three sectors has an angle γ greater than 180°. Note that an angle at a junction cannot be exactly 0 or 180° if the projection is done along a general direction.

A Y-shape junction is said to be *fat* if all three angles are greater than 90°, as shown in Figure 1A, and *slim* otherwise (i.e., if one of the three angles is less than 90°, as shown by α of slim-Y in Figure 1B). Similarly, an arrow-shaped junction is said to be *fat* if the two smaller angles are less than 90° and their sum is greater than 90°, as shown by angles α and β in Figure 1A, and *slim* otherwise (i.e., the sum of two smaller angles is less than 90°, as angles α and β of the type-1 slim-arrow shown in Figure 1B, or the second smallest angle is greater than 90°, as angle β of the type-2 slim arrow shown in Figure 1B).

The following two properties are known (Perkins, 1971; Kanatani, 1986, 1988).

Property 1. For any P-diagram, if one junction is fat, then all of the junctions are fat, and if one junction is slim, then all of the junctions are slim.

Hence, we can shift the term “fat” and “slim” from junctions to P-diagrams. We call a P-diagram *fat* if its junctions are fat, and *slim* if its junctions are slim. In Figure 1, the P-diagram (A) is fat while the P-diagram (B) is slim.

Property 2. A P-diagram can be an orthographic projection of a rectangular parallelepiped if and only if it is fat.

As Perkins (1972) observed, the human vision system is sensitive to whether a P-diagram is fat or slim. Most subjects in Perkins’ experiment interpreted fat P-diagrams as rectangular parallelepipeds and slim P-diagrams as non-rectangular parallelepipeds.

### Pictures With Two Interpretations

#### Pictures Composed of Two P-Diagrams

We want to create pictures that have at least two interpretations as 3D objects, one as rectangular but not symmetric, and the other as symmetric but not rectangular. This cannot be achieved by a single P-diagram, because a rectangular parallelepiped is itself highly symmetric. Hence, we combine two P-diagrams in the following manner.

**FIGURE 2:**
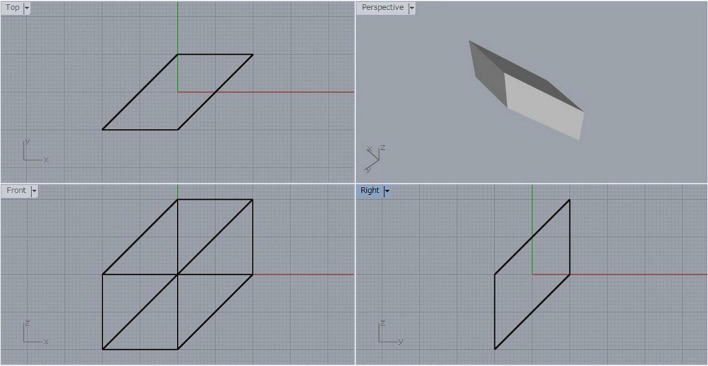
Sheared cube.

Suppose that we have a cube, and we shear it by moving one face in the direction of its diagonal by a distance equal to the length of the diagonal. Let us call the resulting parallelepiped a *sheared cube*. Figure 2 shows a three-view diagram of the sheared cube, where the lower left is the front view, the upper left is the top view, the lower right is the side view, and the upper right is a shaded image of the object viewed from a general viewpoint. The original cube has six square faces, but the sheared cube has only two square faces and the other four faces are parallelograms.

**FIGURE 3:**
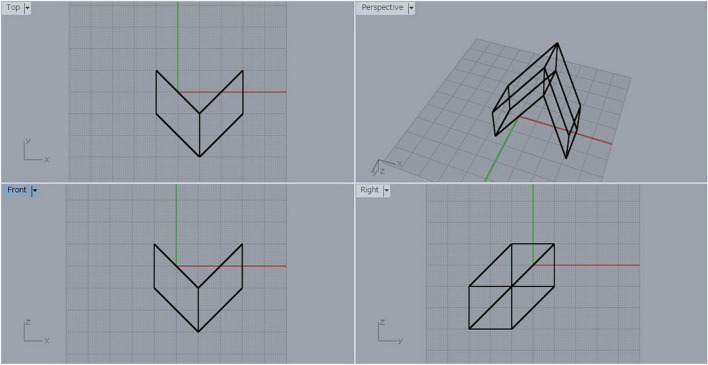
Symmetric object composed of a sheared cube and its mirror-symmetric counterpart.

Let F denote one of the square faces of the sheared cube. We generate the mirror-symmetric object of the sheared cube with respect to the mirror containing the face F, and consider the object composed of the original sheared cube and its mirror-symmetric counterpart. Let us name this object the *object SS* (SS is an abbreviation of sheared cube and sheared cube). The three-view diagram for object SS is shown in Figure 3. This is a mirror-symmetric object with respect to the plane containing the face F. Therefore, any projection of this object can be interpreted as a symmetric but non-rectangular object.

**FIGURE 4:**
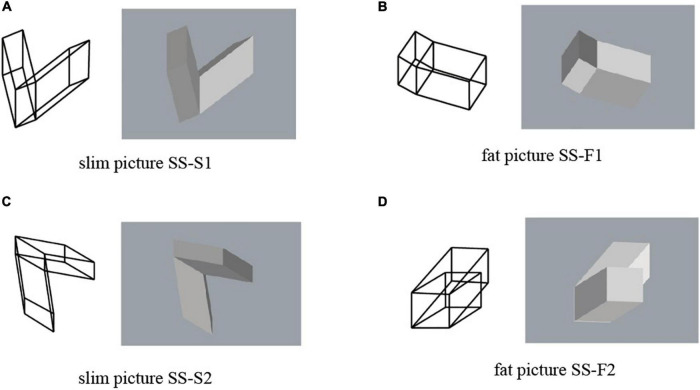
Pictures of object SS: **(A)** picture SS-S1; **(B)** picture SS-F1; **(C)** picture SS-S2; **(D)** picture SS-F2.

Four projected pictures of object SS are shown in Figure 4. They are orthographic projections. We use a pair of the wireframe picture and the shaded picture. This is because the shaded picture cannot represent the hidden part of the object, while the wireframe picture creates visual flip phenomenon known as the Necker cube illusion (Gregory, 1970). In the experiments, we always show pictures in pairs to the subjects. The pictures in Figures 4A,C consists of two slim P-diagrams. We name them SS-S1 and SS-S2, as shown in this figure. On the other hand, the pictures in Figures 4B,D consist of two fat P-diagrams. We name them SS-F1 and SS-F2.

#### Pictures Composed of Three P-Diagrams

**FIGURE 5:**
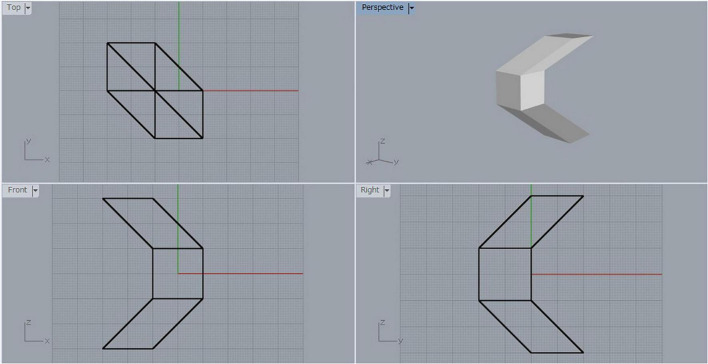
Object SCS composed of a cube and two sheared cubes.

We create a new object from object SS by inserting a cube between the two sheared cubes and gluing them at the square faces. The resulting object is shown in Figure 5 as a three-view diagram. This object is plane symmetric with respect to the plane passing through the center of the cube. We name this object the object SCS (SCS is an abbreviation of sheared cube, cube and sheared cube). This object is symmetric, and consequently any projected picture has an interpretation as a symmetric object. Moreover, this object contains a cube, and consequently any projected picture has a fat P-diagram.

**FIGURE 6:**
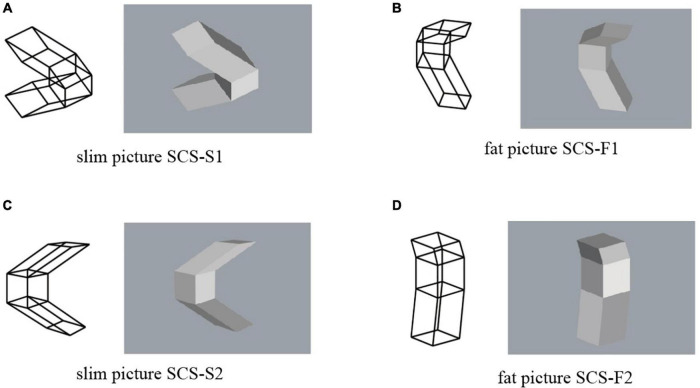
Pictures of object SCS: **(A)** picture SCS-S1; **(B)** picture SCS-F1; **(C)** picture SCS-S2; **(D)** picture SCS-F2.

Examples of projected pictures of object SCS are shown in Figure 6. The pictures in Figures 6A,C are composed of a fat P-diagram corresponding to the cube and two slim P-diagrams; we name them SCS-S1 and SCS-S2. The pictures in Figures 6B,D have three fat P-diagrams corresponding to the center cube, and the two side parallelepiped; we name them SCS-F1 and SCS-F2. SCS-S2 is a perspective projection and all the others are orthographic projections.

### Methods

#### Procedure

The 16 subjects were welcomed and invited to sit. They were told that the experiment did not mean to test their intelligence, personality or emotions, but that they only focus on attention for the experimenter was the way they perceived the target objects on the questionnaire. They were specified that the test respects anonymity. The subjects were then asked to complete the questionnaire. Every subject was explained to tick boxes on the left side of the page to give their answers concerning the wireframe picture, and to tick the boxes on the right side of the page to answer the questions in relation to the shaded picture. The subjects were not given any suggestions but the experimenter let them work independently. In fact, the experimenter only gave clarifications concerning the task if he eventually was asked. No limited time was given to the subjects and they were also free to change their mind and tick another box if that was the case.

They did not have any special knowledge about this topic, and we did not explain the meanings of the terms such as “rectangular” and “symmetric.” We tried to collect their naïve responses when they saw the pictures.

#### Experiment 1

The four pairs of pictures of object SS in Figure 4 were used in this experiment; each pair consists of a wireframe diagram and a shaded image. The subjects were asked to select one answer from among several pre-specified answers to each question for each of the wireframe diagrams and the shaded images. The questions and the pre-specified answers are as follow:

Question SS1. Is it mirror symmetric?
  Answers to be selected: (1) Symmetric, (2) Non-symmetric, or (3) Unknown.

Question SS2. Does it contain rectangular parallelepipeds?
  Answers: (1) No, (2) One part is rectangular, or (3) Both are rectangular.

#### Experiment 2

The four pictures of object SCS in Figure 6 were used in this experiment. Each picture consists of a wireframe diagram and a shaded image. The four pictures were given to the same subjects as in Experiment 1. The subjects were asked to select one answer from among several pre-specified answers to each question for each of the wireframe diagrams and the shaded images. The questions and the pre-specified answers are as follow:

Question SCS1. Is the center part rectangular?
  Answers: (1) Rectangular, (2) Non-rectangular, or (3) Unknown.

Question SCS2. Are the two side parts rectangular?
  Answers: (1) Both are rectangular, (2) One is rectangular and the other is not, (3) Both are non-rectangular, or (4) Unknown.

Question SCS3. Is the whole structure mirror symmetric?
  Answers: (1) Symmetric, (2) Non-symmetric, or (3) Unknown.

## Results

### Results for the Object SS

**FIGURE 7:**
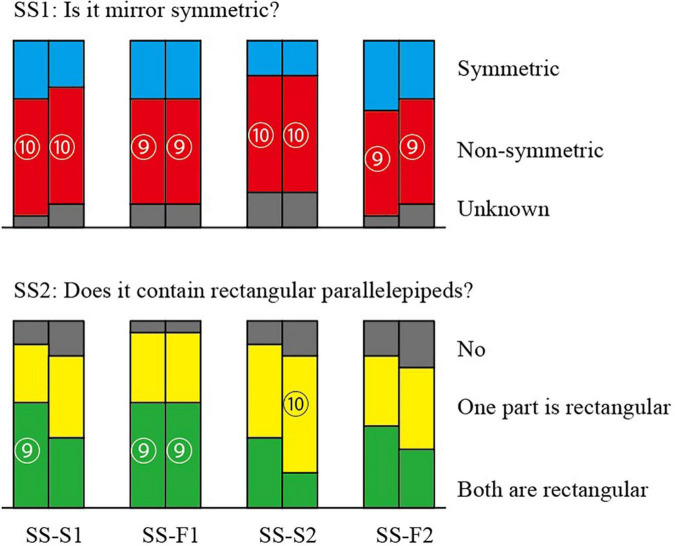
Answers to the questions for the pictures SS’s.

The answers to Questions SS1 and SS2 are summarized in Figure 7. The upper part is for SS1 and the lower part is for SS2. Each pair of adjacent columns summarizes the answers to each pair of pictures. The left column corresponds to the wireframe pictures and the right column corresponds to the shaded picture. The four pairs of columns correspond, in the order from left to right, to the pictures SS-S1, SS-F1, SS-S2, and SS-F2. The number of subjects who chose the same answer is represented by the height of the column with the same color. The numbers in the circles represent the number of subjects who chose the associated answer. We represented those numbers only for significant answers.

We carried out the statistical test in the following way. There are three prespecified answers, and the subjects choose one of them. The null hypothesis is that each answer is chosen independently in equal provability. Let *p*(*k*) be the provability that the same answer is chosen by *k* or more subjects and the other subjects choose any of the other two answers. Then,


$$p\left(k\right)=\sum_{i=k}^{n}\left(\begin{array}{c} n\\ i \end{array}\right)\left(\sum_{j=0}^{n-i}\left(\begin{array}{c} n-i\\ j \end{array}\right)\right)/3^{n}$$

where *n* = 16, and $\left(\begin{array}{c} m\\ l \end{array}\right)$ denotes the number of combinations of choosing *l* subjects from *n* subjects. Substituting *k* = 11 and 9 in this expression, we have *p*(11) = 0.00404 and *p*(9) = 0.04996. Hence, the answer is significant with 1% significance level if eleven or more subjects choose it, and it is significant with 5% significance level if nine or more subjects choose it. In Figure 7, the numbers of subjects are represented in circles only for the significant answers with 5% significance level.

For Question SS1, significantly many subjects answered that it is not mirror symmetric for all the four pairs of the pictures, although all the pictures are the projections of the same symmetric 3D object SS shown in Figure 3.

For Question SS2, on the other hand, many subjects answered that at least one part is rectangular. Mathematically, all the four pairs of pictures have interpretations that one part is rectangular but do not have interpretations that both parts are rectangular. However, many subjects answered that both parts are rectangular.

The values *p*(11) and *p*(9) in the above computation might seem a little optimistic because nine out of sixteen may not be so significant from an intuitive point of view. This gap from the intuition might come from the nature of our prespecified set of answers. First, we provided three possible answers instead of two. The case where the same answer is selected by 9 subjects is much rare than the case where it is selected by the same number of subjects from two possible answers. Second, our possible answers include “Unknown” which is selected only when a subject cannot choose a clear answer. However, the null hypothesis places the equal provability to all possible answers. These points should be considered when we read the “significant number of subjects” in our data.

### Results for the Object SCS

**FIGURE 8:**
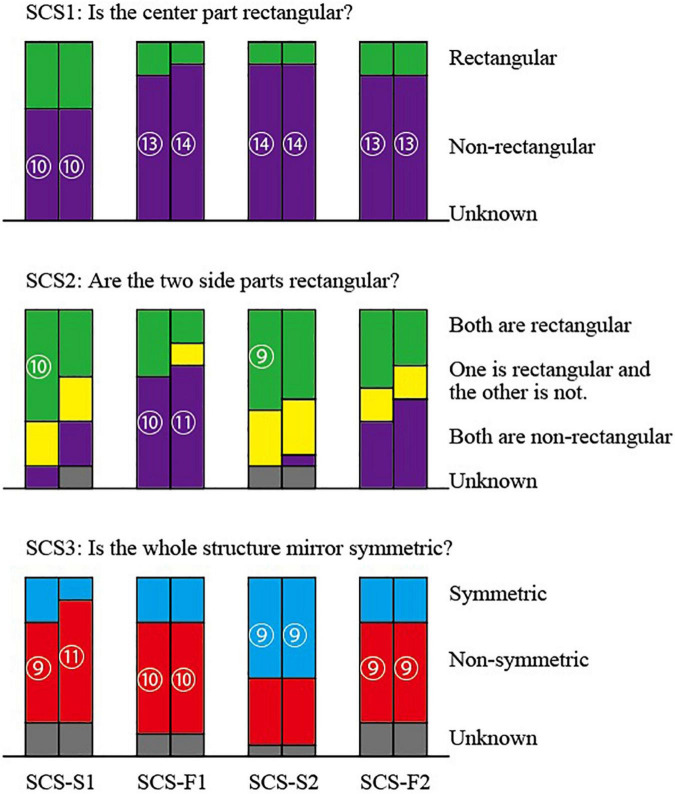
Answers to the question for the pictures SCS’s.

The answers for Object SCS are summarized in Figure 8. The top part corresponds to Question SCS1, the middle part to Question SCS2 and the bottom part to Question SCS3. Each pair of adjacent columns represents the answers of the wireframe picture (left) and the shaded picture (right) for the same posture of the object. The four pairs of columns correspond, in the order from left to right, to the pictures SCS-S1, SCS-F1, SCS-S2, and SCS-F2. Questions SCS1 and SCS3 have three alternative answers and consequently we can apply the statistical test for the ternary distribution; we present the numbers of subjects in the circles only for significant answers with 5% significance level. Question SCS2, on the other hand, has four alternative answers, and hence we cannot apply the ternary distribution. Hence, the numbers of subjects in circles shown in the middle part of the figure are just for reference.

The answers to Question SCS1 have a remarkable tendency that significantly many subjects answered for all the four pairs of pictures that the center part is not rectangular. The other subjects answered that the center is rectangular and no subject chose “Unknown.”

The answers to Question SCS2 show a little complicated behavior. For slim pictures SCS-S1 and SCS-S2, many subjects seem to perceive one or two rectangular parts, while for fat pictures SCS-F1 and SCS-F2, relatively many subjects perceive that neither is rectangular.

To Question SCS3, significantly many subjects answered “Non-symmetric” for pictures SCS-S1, SCS-F1, and SCS-F2, while a significantly many subjects answered “Symmetric” for picture SCS-S2.

## Discussion

### Discussions for Object SS

Object SS is mirror symmetric, and consequently any projection of the object has an interpretation as a symmetric 3D object. However, significantly many subjects did not perceive symmetry in any of the four pairs of pictures, as shown in the upper part of Figure 7. Thus, a picture of a symmetric 3D structure is not necessarily perceived as being symmetric.

From a mathematical point of view, the slim pictures (i.e., SS-S1 and SS-S2) do not contain a projection of a rectangular parallelepiped. However, this mathematical characteristic was ignored by most of the subjects; more than 80% of the subjects perceived at least one rectangular parallelepiped in these pictures, as shown in the lower part of Figure 7. This result differs from the results of Perkins (1972), who observed that the human vision system is sensitive to the discrimination between correct pictures of rectangular parallelepipeds (i.e., fat diagrams) and pictures that cannot be projections of rectangular parallelepipeds (i.e., slim diagrams). This difference might result because we use an object consisting of two parallelepipeds while Perkins (1972) used a single parallelepiped.

The basic trend of our results is that, for all four pairs of the pictures, a large number of the subjects perceived one or two rectangular parallelepipeds and considered the whole object to not be symmetric. This supports the claim that the preference for rectangularity is stronger than that for symmetry.

### Discussion for Object SCS

The center part of Object SCS is a cube, and hence any picture has an interpretation that the center part is a cube, which belongs to the rectangular parallelepipeds in a mathematical sense. However, significantly many subjects chose “Non-rectangular” for Question SCS1 (shown at the top of Figure 8). This result might look strange for the first glance, but it revealed that many subjects perceived a cube, but they thought a cube is different from a rectangular parallelepiped and answered “Non-rectangular.” Therefore, this result does not imply that the preference for rectangularity is weak.

The two side parts of Object SCS are slim P-diagrams in pictures SCS-S1 and SCS-S2, and hence cannot be the projections of any rectangular parallelepipeds. However, many subjects answered they are rectangular (the first and the third pairs of columns at the middle of Figure 8). In particular, significantly many subjects answered that both are rectangular for the wireframe diagrams. These results show that the preference for the rectangularity is strong.

The two side parts of Object SCS are fat P-diagrams in pictures SCS-F1 and SCS-F2, and hence each can be the projections of a rectangular parallelepiped. However, significantly many subjects answered that both are non-rectangular to picture SCS-F1 (the second pair of columns in the middle of Figure 8) and many subjects gave the same answer to picture SCS-F2 (the rightmost pair of columns in the middle of Figure 8). This result shows that the human perception is not necessarily consistent with the mathematical correctness. The fat P-diagrams (i.e., correct projection of a rectangular parallelepiped) are perceived as non-rectangular, and the slim P-diagrams (i.e., diagrams that cannot be the projection of a rectangular parallelepiped) are perceived as rectangular.

The results for Question SCS2 also differ from the results of Perkins (1972); he observed that the human vision system is sensitive to correct and incorrect pictures of rectangular parallelepipeds. These differences imply that the human vision system is less sensitive to the correctness of the picture of a rectangular parallelepiped if the object is composed of two or more parallelepipeds.

Object SCS is mirror symmetric, and hence any picture of this object is a correct picture of a symmetric 3D object. Indeed, significantly many subjects answered “Symmetric” for picture SCS-S2 (the third pair of the column at the bottom of Figure 8). However, this picture itself is almost symmetric, because the object is projected in the direction nearly parallel to the plane of the mirror symmetry. Moreover, SCS-S2 is the perspective projection of the object SS while all the others are orthographic projections. In this sense this picture is special.

On the other hand, significantly many subjects answered “Non-symmetric” for pictures SCS-S1, SCS-F1 and SCS-F2. Thus, the pictures of object SCS are perceived to be “non-symmetric” by majority of subjects, unless the picture itself is close to symmetric.

Comparing the answers to Question SCS2 and those to Question SCS3, we can observe another aspect of the difference between the mathematics and the human perception. The same picture can be the projections of many different 3D objects. The pictures of Object SCS can represent a symmetric object whose two side parts are non-rectangular (i.e., the original Object SCS itself). The fat pictures of this object can also represent a 3D object in which one of the side parts is rectangular, but this object is not symmetric. So, from a mathematical point of view, those pictures can be interpreted as symmetric but non-rectangular, and can also be interpreted as non-symmetric but partly rectangular; the symmetry and the rectangularity are mutually conflicting. However, many subjects chose both “Both are non-rectangular” and “Non-symmetric” for pictures SCS-F1 and SCS-F2, and many subjects chose both “Both are or One is rectangular” and “Symmetric” for picture SCS-S2.

This experiment does not suggest the strength of the rectangularity preference as much as the previous experiment.

### Rectangularity Preference and Depth Illusions

#### Room-Size Illusion

Once we accept the preference for rectangularity over symmetry, we can explain some visual phenomena. A typical example is the impression of the size of a room we have when we see certain types of photographs.

When we visit a hotel-reservation website, for example, we often find images of rooms that look bigger than their actual sizes. This phenomenon is well known by photographers; it arises when a picture is taken using a wide-angle lens (Sugihara, 2021). Let us call this the *room-size illusion*.

**FIGURE 9:**
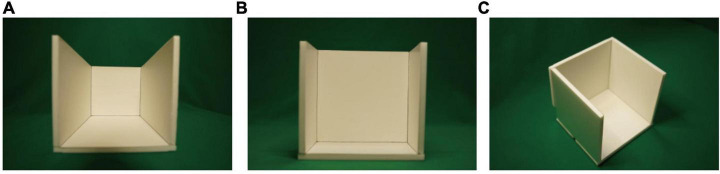
Depth-exaggerated photographs of a cubic room: **(A)** photograph of the cubic room taken by 14 mm focal-length lens; **(B)** photograph of the cubic room taken by 70 mm focal-length lens; **(C)** general view of the cubic room.

Figure 9 shows an example. This is a paper model of a cubic room; the front wall was removed so that photographs could be taken, and the roof was removed to allow more light into the inside. Panel (A) is a photograph of this room taken with a wide-angle lens with a focal length of 14 mm for 35 mm full-frame camera, panel (B) is a photograph taken with a standard lens with a focal length of 70 mm, and panel (C) shows the general appearance of this room. For image (A) or (B), suppose that we fix the viewpoint in front of the center of the image at a particular distance. From a mathematical perspective, the interpretation of the image can be considered to be reconstructing a 3D structure whose projection with respect to the center of the projection at the viewpoint matches the image. There are infinitely many candidates for the 3D structure, among which both a rectangular parallelogram and a square frustum are included. Because of the preference for rectangularity, the rectangular parallelogram will be chosen as the interpretation. For this interpretation, the depth of the room is the same as the width and the height when the viewpoint coincides with the lens center [panel (B) is close to this interpretation], and becomes larger as the viewpoint becomes farther from the photograph [panel (A) demonstrates this case]. Mathematically, panel (A) is a correct projection of a square frustum whose depth is the same as the width and the height of the room. Although a square frustum such as this would be symmetric, the image is not perceived as a square frustum. Thus, the room-size illusion comes from the preference for rectangularity.

There is a famous room-shape illusion named the Ames room (Gregory, 1970, Ninio, 2001). The Ames room is actually non-rectangular and non-symmetric, but it is perceived as being rectangular and hence left-right symmetric. So, the rectangularity preference and the symmetry preference do not conflict each other; both of them together may contribute to create the Ames room illusion.

#### Apparent Deformation of Solid Objects Caused by Viewpoint Change

**FIGURE 10:**
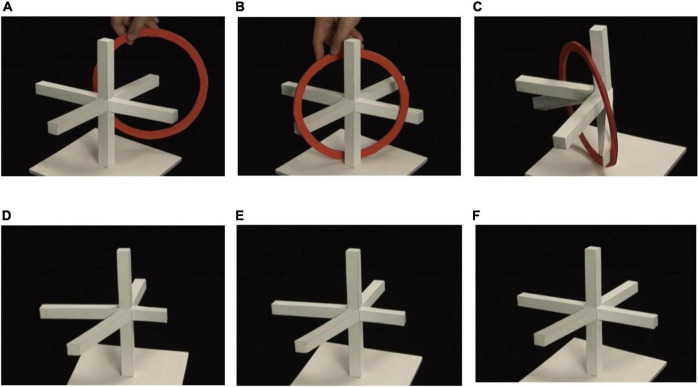
Impossible motion illusion: “Four Perches and a Ring”: **(A)** pole and four perches; **(B)** impossible motion of a ring; **(C)** true shape of the object; **(D–F)** continuous deformation of the object shape.

We sometimes observe the impression of continuous deformation of a solid object when we move the viewpoint continuously. Figure 10 shows an example of such an object. This object creates impossible motion illusion (Sugihara, 2005, 2014). When we see this object from a special viewpoint, as shown in (A), the object appears to be composed of a vertical column and four horizontal bars meeting at right angles. However, we can hang a flat ring over this object in a manner as shown in (B), where the ring passes behind the column but passes in front of all the four bars. This motion of the ring appears to be impossible. The true shape of the object is as shown in (C); the four bars all extend toward backward, and consequently the motion of the ring is physically possible. The truth is that some of the bars are not rectangular, but appear to be rectangular from the special viewpoint.

In exhibition, we first show this impossible motion, next reveal the trick by rotating the object, then remove the ring, and finally rotate the object in reverse direction to come back to the initial posture. Then, many observers report that the object appears to deform continuously. This visual effect does not happen much while the ring is hung, but it happens strongly after the ring is removed. The sequence of panels (D–F) show snapshots of the object when it is rotated toward the initial posture. The two rods that extend obliquely downward appear to gradually rise up to the horizontal level.

This visual phenomenon can be explained by the preference for rectangularity. The rods are parallelepipeds. If they generate slim P-diagram in the image plane, they are perceived as nearly correct shapes of the rods, as is the case when we see panel (C). If they generate fat P-diagram, on the other hand, they are perceived as rectangular parallelepipeds, which are not the true shapes. Different viewpoints generate different fat P-diagrams, which in turn create interpretations of different rectangular parallelepipeds (recall Property 2). Consequently they appear to deform when the viewpoint moves.

**FIGURE 11:**
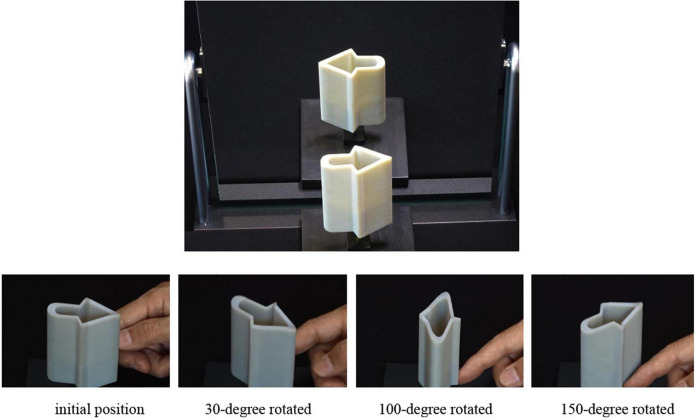
Continuous deformation of the “right-facing arrow”: an arrow that change the direction in a mirror (top), and continuous deformation of the right-facing arrow that faces toward right again when it is rotated by 180° (bottom).

Another example is shown in Figure 11. The top photograph shows the object and its image reflected by a vertical mirror. The behavior seems impossible because the right-facing arrow turns toward the left in the mirror. This object is an example of the ambiguous cylinders (Sugihara, 2015, 2016), which have two quite different appearances and it is difficult to believe that they come from the same object. When we rotate this object around a vertical axis, the appearance changes as shown by a sequence of snap shots at the bottom. The object initially faces toward the right, and it faces toward the right again when it is rotated by 180°. Moreover, the object seems to deform continuously during the rotation.

The true shape of this object is a cylinder, but the top curve is not planar; it undulates up and down. However, the top curve looks as if it is a section of the cylinder obtained by cutting by a horizontal plane. This visual effect seems to come from the preference of rectangularity because of the following reason. The bottom of the cylinder was cut by the same curve as the top curve, and consequently the vertical length of the side is the same wherever we measure it. This gives us an impression that the cylinder has a uniform height, and our brains consider that the top curve is on the plane perpendicular to the axis of the cylinder.

Note that this object is line symmetric with respect to the vertical line. This is the reason why the 180-degree rotation creates the same appearance as the initial. However, it is not easy to understand this symmetry. If we could understand the symmetric nature of this object, we might be able to understand the true shape. But this does not happen; instead we consider that the top curve is perpendicular to the axis. Thus, this is another example of visual phenomenon, in which the preference of rectangularity is stronger than that of symmetry.

## Data Availability Statement

The raw data supporting the conclusions of this article will be made available by the authors, without undue reservation.

## Ethics Statement

Ethical review and approval was not required for the study on human participants in accordance with the local legislation and institutional requirements. The patients/participants provided their written informed consent to participate in this study.

## Author Contributions

KS and BP contributed to the conception, study design, and analysis. KS prepared the materials. BP performed the experiments. Both authors critically reviewed the manuscript and contributed to the editing of the final draft.

## Conflict of Interest

The authors declare that the research was conducted in the absence of any commercial or financial relationships that could be construed as a potential conflict of interest.

## Publisher’s Note

All claims expressed in this article are solely those of the authors and do not necessarily represent those of their affiliated organizations, or those of the publisher, the editors and the reviewers. Any product that may be evaluated in this article, or claim that may be made by its manufacturer, is not guaranteed or endorsed by the publisher.

## References

[B1] Clowes M. B. (1971). On seeing things. *Artif. Intell.* 2 79–116. 10.1016/0004-3702(71)90005-1

[B2] Gregory R. L. (1970). *The Intelligent Eye.* London: Weidenfeld & Nicolson.

[B3] Hertzmann A. (2020). Why do line drawings work? A realism hypothesis. *Perception* 49 439–451. 10.1177/0301006620908207 32126897

[B4] Hidaka S. Takahashi K. (2021). Why does the Necker cube appear as a three-dimensional shape? (in Japanese). *Bull. Jpn Cogn. Sci. Soc.* 28 25–38. 10.31234/osf.io/cxgkd

[B5] Hoffman D. D. (1998). *Visual Intelligence: How We Create What We See.* New York, NY: W. W. Norton & Company.

[B6] Kanatani K. (1986). The constraints on images of rectangular polyhedrals. *IEEE Trans. Pattern Anal. Mach. Intell.* 8 456–463. 10.1109/tpami.1986.4767809

[B7] Kanatani K. (1988). Constraints on lengths and angles. *Comput. Vision Graph. Image Process.* 41 28–42. 10.1016/0734-189X(88)90115-6

[B8] Marr D. (1982). *Vision: A Computational Investigation into the Human Representation and Processing of Visual Information.* New York, Ny: W. H. Freeman and Company.

[B9] Michaelsen E. (2014). Gestalt algebra—a proposal for the formalization of gestalt perception and rendering. *Symmetry* 6 566–577. 10.3390/sym6030566

[B10] Michaelsen E. Muench D. Arens M. (2013). Recognition of symmetry structure by use of gestalt algebra. *Proc. CVPR* 2013 206–210. 10.1109/CVPRW.2013.37

[B11] Ninio J. (2001). *The Science of Illusions (English translation).* Ithaca: Cornell University Press.

[B12] Perkins D. N. (1971). *Cubic Corners, Oblique Views of Pictures, the Perception of line Drawings of Simple Space Forms. Geometry and the Perception of Pictures: three Studies. Technical Report no. 5.* Cambridge: Graduate School of Education, Harvard University.

[B13] Perkins D. N. (1972). Visual discrimination between rectangular and nonrectangular parallelepipeds. *Percept. Psychophys.* 12 293–331. 10.3758/BF03205849

[B14] Perkins D. N. (1973). Compensating for distortion in viewing pictures obliquely. *Percept. Psychophys.* 14 13–18. 10.3758/bf03198608 9522677

[B15] Sawada T. Li Y. Pizlo Z. (2014). Detecting 3-D mirror symmetry in a 2-D camera image for 3-D shape recovery. *Proc. IEEE* 102 1588–1606. 10.1109/jproc.2014.2344001

[B16] Sugihara K. (1986). *Machine Interpretation of Line Drawings.* Cambridge, MA: The MIT Press.

[B17] Sugihara K. (2005). A characterization of a class of anomalous solids. *Interdiscip. Inf. Sci.* 11 149–156. 10.4036/iis.2005.149

[B18] Sugihara K. (2014). Design of solids for antigravity motion illusion. *Comput. Geom. Theory Appl.* 47 675–682. 10.1016/j.comgeo.2013.12.007

[B19] Sugihara K. (2015). Ambiguous cylinders: a new class of impossible objects. *Comput. Aided Drafting Des. Manuf.* 25 19–25.

[B20] Sugihara K. (2016). Anomalous mirror symmetry generated by optical illusion. *Symmetry* 8:21. 10.3390/sym8040021

[B21] Sugihara K. (2021). True views from depth-exaggerated images. *Proc. Int. Disp. Workshops* 28 1038–1041.

[B22] Valley P. A. C. Martin R. R. Suzuki H. (2004). “Making the most of using depth reasoning to label line drawings of engineering objects,” in *Proceedings of the 9th ACM Symposium on Solid Modeling and Applications SM’04*, eds Elber G. Patrikalakis N. Brunet P. (Genoa: Eurographics Association) 191–202.

